# The antihyperglycemic and hypolipidemic effects of *Ribes khorassanicum* hydro-ethanolic extract co-administration in type 2 diabetic patients: A randomized double blind placebo controlled trial

**DOI:** 10.22038/AJP.2021.51446.2676

**Published:** 2022

**Authors:** Shahriar Dashti, Mousa-Al-Reza Hadjzadeh, Ahmad Ghorbani, Masoud Mohebbi, Zahra Gholamnezhad

**Affiliations:** 1 *Department of Internal Medicine, Faculty of Medicine, Mashhad University of Medical Sciences, Mashhad, Iran*; 2 *Division of Neurocognitive Sciences, Psychiatry and Behavioral Sciences Research Center, Mashhad University of Medical Sciences, Mashhad, Iran*; 3 *Pharmacological Research Center of Medicinal Plants, Mashhad University of Medical Sciences, Mashhad, Iran*; 4 *Metabolic Syndrome Research Center, Mashhad University of Medical Sciences, Mashhad, Iran*; 5 *Department of Physiology, Faculty of Medicine, Mashhad University of Medical Sciences, Mashhad, Iran*; 6 *Applied Biomedical* * Research Center, Mashhad University of Medical Sciences, Mashhad, Iran*

**Keywords:** Diabetes mellitus, Ribes khorassanicum, Hyperglycemia, Hyperlipidemia, Co-supplementation

## Abstract

**Objective:**

The present randomized clinical trial assessed the antihyperglycemic and hypolipidemic effects of hydro-ethanolic extract of *Ribes khorassanicum*.

**Materials and Methods:**

Eighty type 2 diabetic patients were randomly allocated to placebo or intervention groups and respectively received placebo or extract capsules (700 mg, bid) beside their conventional medication for 3 months. Patients' blood pressure and blood levels of fasting blood glucose (FBS), glycosylated hemoglobin (HbA1c), 2 hr postprandial glucose (2hPPG), triglyceride, total cholesterol, low-density lipoprotein cholesterol (LDL-C), and high-density lipoprotein cholesterol (HDL-C) were measured at the beginning of the study and after 3 months of treatment. For determination of plant safety, liver enzymes (SGOT and SGPT) and kidney function (in terms of urea, creatinine, and microalbumin levels) were assessed and patients were asked to report adverse effects.

**Results:**

The *R. khorasanicum* hydro-ethanolic extract supplementation significantly decreased the levels of FBS, total cholesterol, triglyceride, and LDL-C in the extract group compared to the placebo group (p<0.05-p<0.01). However, 2hPPG, HbA1c, HDL-C, SGOT, SGPT, urea, creatinine, and urine microalbumin values were not significantly different between the placebo and the extract groups. No adverse effects were reported by the patients.

**Conclusion:**

Co-supplementation of diabetic patients with *R. khorasanicum* extract ameliorated hyperglycemia and hyperlipidemia without causing any adverse effects; therefore, the plant extract may be recommended as a complementary therapy to improve diabetes-induced metabolic disturbances.

## Introduction

Diabetes mellitus is a common metabolic disease with long-term disabling complications which decrease patients' quality of life while increasing the risk of various diabetes-associated morbidities and even mortality (Baena-Diez et al., 2016 ▶). Diabetes is regarded as one of the major health problems worldwide because of its high prevalence (463 million by 2019) which is estimated to increase by 25% in 2030, and costly treatment (Saeedi et al., 2019 ▶). In type 2 diabetic patients, chronic hyperglycemia and metabolic disturbance lead to macro and micro vascular damages which induce cardiovascular dysfunction, nephropathy, retinopathy, and neuropathy. Therefore, complications including heart attacks, stroke, kidney failure, blindness, lower limb amputation, and premature death are common in diabetic patients with uncontrolled metabolic homeostasis (Hippisley-Cox and Coupland, 2016 ▶; Karuranga and Duke, 2018 ▶). Among such consequences, renal disease and cardiovascular complications effectively reduce the quality of life and lifespan of patients (Garg et al., 2020 ▶). Although several medications have been prescribed for management of diabetes and glycemic control, their long-term efficacy and safety are under debate. Therefore, combination and adjunct therapy with new drugs and herbal product have been proposed for achieving glycated hemoglobin (HbA1C) target level below 7% and reducing diabetes complications (Garg et al., 2020 ▶; Governa et al., 2018 ▶).^. ^

In recent decades, medicinal plants have been examined for antihyperglycemic, antihyperlipidemic and cardio/reno-protective activities in experimental and clinical studies (Fatehi-Hassanabad et al., 2005 ▶; Sotoudeh et al., 2019 ▶). “*Ghareghat*” (Persian name) with the scientific name *Ribes khorasanicum* (*R. khorasanicum*) is a native and endemic medicinal plant spices in north of Khorasan Razavi province, Iran, which belongs to the family of Grossulariaceae (Saghafi and Assadi, 1996 ▶). In Iran, the genus *Ribes* has four identified taxonomic spices including the *Vaccinium arctostaphylos *L.,* Ribes biebersteinii* Berland., *Ribes khorasanicum* F. Saghafi and Assadi, *Ribes orientale* Desf. (Joharchi and Amiri, 2012 ▶). *R. khorasanicum* shrubs grow land stretches from Kalat to Dargaz, Laein to Ors Nahalestan, and on heights of Hezar Masjed. The locals use *R. khorasanicum *for treatment of gastrointestinal complications and lowering blood pressure and blood glucose. In traditional medicine, Ghareghat’s fruit seeds and leaves are recommended as a herbal remedy for diabetes, hyperlipidemia and hypertension (Mir Heidar, 2004 ▶), and few experimental studies have been performed to study these effects (Adibi et al., 2007 ▶; Gholamnezhad et al., 2021 ▶; Hamounpeima et al., 2019 ▶). Phytochemical evaluation of the plant flowers and ripe and unripe fruits has revealed the presence of phenolic, flavonoid, saponin, tannin, and alkaloid compounds with effective antimicrobial properties (Adibi et al., 2007 ▶). Recently total flavonoid, phenolic, anthocyanin, and soluble and insoluble hydrocarbons contents, as well as the antioxidant activity of *R. khorasanicum* were determined. The main antioxidant phenolic compound in the *R. khorasanicum* is anthocyanin (Taghavizadeh Yazdi et al., 2018 ▶). Several studies have demonstrated the protective roles of flavonoids and anthocyanins against diabetes, insulin resistance, hyperlipidemia, cardiovascular disease, and cancer (Belwal et al., 2017 ▶; Latti et al., 2009 ▶; Turrini et al., 2017 ▶). 

The anti-diabetic and antihyperlipidemic effects of other taxonomic identified spices of Ghareghat were demonstrated in both animal experimental models and human studies (Zolfaghari et al., 2015 ▶). The antihypertensive and metabolic effects of Caucasian whortleberry or *Vaccinium arctostaphylos* (*V. arctostaphylos*) have been demonstrated in animal and human studies. Treatment of diabetic rats with* V. arctostaphylos *fruit ethanolic extract showed antioxidant, anti-hypertriglyceridemic, and antihyperglycemic effects, which were related to its stimulatory effect on the pancreatic insulin and myocardial glucose transporter 4 genes expression as well as its inhibitory action on α-glucosidase activity (Feshani et al., 2011 ▶). Moreover, in several clinical trials, the antihyperglycemic and anti-hyperlipidemic properties of *V. arctostaphylos* fruit and leaf extract were indicated (Kianbakht et al., 2013 ▶; Kianbakht et al., 2014 ▶; Soltani et al., 2014 ▶). However, to the best of our knowledge, there is no clinical trial evaluating the anti-diabetic properties of *R. khorasanicum*. Therefore, the aim of this study was to investigate the anti-hyperglycemic and antihyperlipidemic effects of *R. khorasanicum* hydro-ethanolic extract in diabetic patients.

## Materials and Methods

**Preparation of extracts**

The dried plant fruits of *R. khorasanicum* were purchased from a local herb market in August 2018. The plant’s specimen was identified by botanists in the herbarium of Mashhad School of Pharmacy (specimen number:13233). To prepare the hydro-ethanolic extract, 100 g of chopped *R. khorasanicum* dried fruit was mixed with 2800 ml of 50% ethanol for 72 hr at 40°C. Then, the solvent (ethanol+water) was totally removed by rotary evaporation under reduced pressure, resulting in an extraction yield of 34.11% (W/W). This process was then repeated several times during the study. The dried fruit and the extract were stored at 4°C in a closed dark container. 

**Preparation of the placebo and** ***R. khorasanicum*** **fruit extract capsules**

Avicel microcrystalline cellulose (Ph-102) was used for granulation and powdering the extract and as placebo powder. For preparation of the final dry extract powder, the extract and excipient were mixed at 1:1 w/w ratio. Gelatin capsules (700±50 mg) containing placebo or extract powder were prepared using a hand-operated capsule-filling machine (Scientific Instruments and Technology Corporation, USA). Each *R. khorasanicum* hydro-ethanolic extract powder capsule contained 350 mg of the dried extract powder. The *R. khorasanicum* and placebo capsules were identical in all aspects. 

**Standardizing the** ***R. khorasanicum*** **extract**

The extract was standardized based on its phenolic content by Folin-Ciocalteu method. Briefly, a sample (20 µl) of the extract (10 mg/ml) or gallic acid (as standard) was added to Folin-Ciocalteu reagent (100 µl) and sodium carbonate (300 µl, 1 mol/l). Then, volume of the mixture was adjusted to 2 ml using deionized water and the optical absorbance was measured after 2 hr at 765 nm, using spectrophotometer (Biotek, USA). The standard curve of gallic acid (0, 50, 100, 150, 250, and 500 mg/l) was plotted and the content of total phenols in the extract was expressed as milligram of gallic acid equivalents (Hosseini et al., 2017 ▶).

**Subjects' enrolment and allocation**

**Figure 1:**
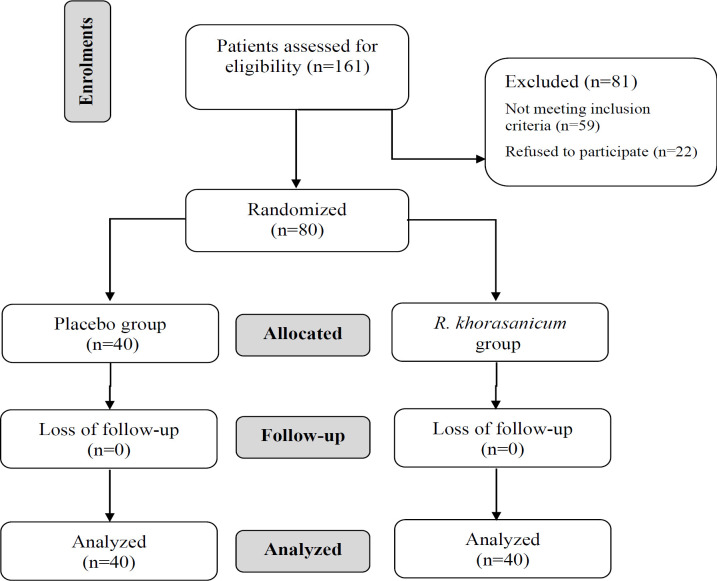
The consort flow diagram of the study

In the present double-blinded clinical study, diabetic patients of both sexes referring to Diabetes Center of Imam Reza Hospital, Mashhad University of Medical Sciences (Mashhad, Iran) were enrolled from September 09, 2018 to January 25, 2020. For calculating sample size the statistical parameters of previous studies were considered (Kianbakht and Hashem-Dabaghian, 2019 ▶; Mohtashami et al., 2019 ▶). Patients were assessed for eligibility based on the following inclusion criteria: Age between 35-60 years, being diagnosed with diabetes for at least 6 months (FBS≥126 mg/dl, Blood sugar 2 hr post-prandial (2hPPG) ≥200 mg/dl and HbA1c≥6.5%), who were receiving oral anti-diabetic drug. The exclusion criteria were as follows: Patients with cardiac, renal, and hepatic diseases, patients who had concomitant infections, patients with insulin injection or smoking, pregnant and breastfeeding women, patients with recent (in the last month) and concurrent use of herbal medications, aspirin, anticoagulant drugs and those who reported any side effect due to the study intervention. From 161 patients, 59 patients were excluded and 22 patients refused to participate. The remaining 80 patients were randomly assigned to the placebo or *R. khorasanicum* extract intervention groups using block randomization by computer-generated random number Table (Figure 1). The patients were asked to sign a written informed consent form before participation in the study and they were assured about confidential process of recording and publishing of their data. The participants were asked to take placebo or extract capsules every 12 hr/day alongside their current medications. This clinical trial was registered in the Iranian Registry of Clinical Trail (IRCT ID: IRCT2015110219739N1). 

**Biomedical evaluation**

At the start of the study (i.e. baseline) patients were asked about demographic data. Moreover, at the beginning of the study and after the 3-month intervention period, biochemical parameters including blood levels of FBS, 2hPPG, HbA1c, total cholesterol, triglyceride, LDL-C, and HDL-C were analyzed, and blood pressure (BP) measurement was done. Biochemical assessment was done using auto-analyzer (Hitachi 917, Japan) and ambulatory blood pressure monitoring for BP measurement. For determination of extract safety, the patients were asked to report any adverse effects occurring during the study, and at the beginning and at the end of the intervention, liver enzymes (SGOT and SGPT) and kidney function (in terms of urea, creatinine, and microalbumin level) were assessed. 

**Statistical analysis**

Statistical analyses were performed using SPSS 20 software. For data analysis, chi-square test and student t-test were carried out and significance level was set at p<0.05.

## Results

**Content of phenolic compounds in the extract**

The content of phenolic compounds in the extract of *R. khorasanicum* was 54 mg gallic acid equivalent per gram of the extract. Considering the percentage of solid residue obtained through plant extraction (34.11%), each gram of *R. khorasanicum* contained 18.36 mg phenolic compounds (gallic acid equivalent). Since, each 700 mg-capsule contained 350 mg dried extract of *R. khorasanicum*, the equivalent phenolic compounds in each capsule was 18.9 mg.

**Lipid, glucose and blood pressure profile of the study population**

The placebo and extract groups were matched for baseline demographic data, including gender, age, education, anti-diabetic medication, diabetes duration, body mass index (BMI) and blood pressure. There were no significant differences between groups in these parameters at baseline (p>0.05 for all) (Table 1). In addition, before intervention data analysis showed that there were no significant differences in biomedical parameters including blood levels of FBS, 2hPPG, HbA1c, total cholesterol, triglyceride, LDL-C, and HDL-C, SGOT, SGPT, urea, creatinine, and urine microalbumin level between the placebo and extract groups (p>0.05 for all).

After intervention, with in groups evaluation showed that in the extract group, the levels of FBS, 2hPPG, HbA1c, total cholesterol, triglyceride, and LDL-C, as well as diastolic and mean BP values were significantly decreased compared to baseline (p<0.001 for all). Moreover, in the placebo group the FBS, 2hPPG, total cholesterol, diastolic and mean BP values were significantly decreased compared to baseline (p<0.01-p<0.001) (Tables 2).

**Table 1:** Baseline characteristics of the study participants

**Variable**	**Placebo group (N=40)**	***Ribes khorasanicum*** **group (N=40)**	**p value**
**Age(year)**	47.1±8.21	49.07±7.12	0.25**
			
**Gender**	N (%)	N (%)	
**Female**	21 (52.5)	25 (62.5)	0.49*
**Male **	19 (47.5)	15 (37.5)	
			
**BMI (kg/m** ^2^ **)**	29.77±4.7	29.08.8±3.58	0.47**
**BMI**	N (%)	N (%)	0.87 *
**Normal **	3 (7.5)	3 (7.5)	
**Overweight **	23 (57.5)	26 (65)	
**Obese **	14 (35)	11 (27.5)	
			
**Education**	N (%)	N (%)	0.57*
**Illiterate **	3 (7.5)	4 (10)	
**Primary School**	22 (55)	26 (65)	
**Secondary School**	10 (25)	8 (20)	
**University **	5 (12.5)	2 (5)	
			
**Oral anti diabetic medication**	N (%)	N (%)	0.83*
**Metformin**	16(40)	19 (47.5)	
**Other**	24 (60)	21 (52.5)	
			
**Diabetes duration (year)**	5.25±3.61	6.7±4.9	0.14**
**SBP (mm Hg)**	130±12.9	129.75±10.12	0.92**
**DBP (mm Hg)**	72.9±6.97	71.5±5.45	0.33**
**Mean BP (mm Hg)**	91.9±8.3	90.91±6.36	0.55**

Data are presented as percentage and Mean±SD (standard deviation). BMI=body mass index; SBP=systolic blood pressure; DBP=diastolic blood pressure. *chi-squared test and **independent t-test were used to compare the two groups at baseline.

**Table 2:** Clinical parameters at baseline and after 90-day intervention

**Differences between groups (p-value )**		**Placebo**				***Ribs khorassanicum***				**Parameter**	
**After Int.**		**Before Int.**		**p-value**	**Day 90**	**Baseline**		**p-value**	**Day 90**	**Baseline**		
0.003		0.67		0.004	159.1**±**30.7	173.77±41.7		<0.0001	140.77**±**22.9	177.63±39.79		**FBS (mg/dl)**
0.281		0.822		0.0007	233.72**±**66	262.12±71.38		<0.0001	219.62**±**48.97	258.58±69.07		**2hPPG (mg/dl)**
0.42		0.88		0.1072	8 ±1.06	8.24±1.14		0.0014	7.81 ±1.08	8.20±1.31		**HbA1c (%)**
0.046		0.98		0.6446	161.65±57.92	165.57±64.18		0.0011	137.9±46.15	160.75±70.01		**TG (mg/dl)**
0.036		0.357		0.0006	167.1±32.32	178.12±33.07		<0.0001	153.65±23.42	185.62±39.10		**TC (mg/dl)**
0.003		0.84		0.622	108.82±32.13	110.62±33.98		0.0002	89.24±25.38	114.62±35.41		**LDL-C (mg/dl)**
0.528		0.445		0.3442	37.62±5.85	38.85±5.65		0.4708	36.82±5.43	37.8±6.54		**HDL-C (mg/dl)**
0.119		0.348		0.6859	25.07±12.31	22.80±6.38		0.5681	21.55±7.01	22.47±7.40		**SGOT (U/l)**
0.899		0.679		0.8971	25.55±10.23	25.85±10.44		0.6846	25.85±10.85	26.87±11.61		**SGPT (U/l)**
0.658		0.377		0.3329	17.12±3.61	18.07±4.99		0.6580	17.75±8.15	17.07±5.08		**Urea (mg/dl)**
0.312		0.06		0.8176	0.94±0.11	0.95±0.17		0.156	0.97±0.14	1.02±0.19		**Creatinine (mg/dl)**
0.838		0.94		0.6902	23.17±14.46	21.83±15.52		0.9383	22.4±19.12	22.09±15.85		**MAU (mg/l)**
0.78		0.92		0.0648	124.87±11.5	130±12.9		0.0481	125.5±8.75	129.75±10.12		**SBP (mmHg)**
0.23		0.33		0.0027	68.12±6.76	72.9±6.97		<0.0001	66.5±5.33	71.5±5.45		**DBP (mmHg)**
0.56		0.54		0.0079	87.04±11.5	91.9±8.3		0.0008	86.16±5.77	90.91±6.36		**Mean BP (mmHg)**

Data are presented as percentage and Mean±SD (standard deviation). Independent samples t-test was used for statistical analysis between groups and dependent sample t-test was used for statistical analysis within groups. Int=intervention; FBS=Fasting blood glucose; 2hPPG=2 hours postprandial; TG=Triglyceride; TC=Total cholesterol; MAU=Urine Microalbumin; SBP=Systolic blood pressure; DBP=Diastolic blood pressure.

**Figure 2:**
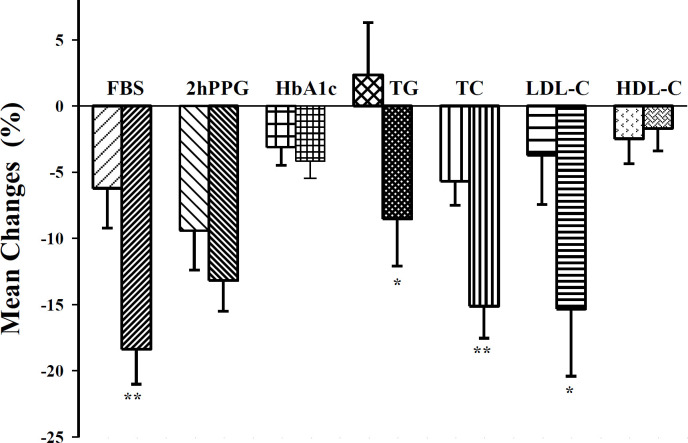
Mean changes (%) of FBS (Fasting blood glucose), 2hPPG (2 hours postprandial), TG (Triglyceride), TC (total cholesterol), LDL-C (low-density lipoprotein cholesterol) and HDL-C (high-density lipoprotein cholesterol) of placebo (medium filled bars) and *Ribes khorasanicum* group (fine filled bars). *p<0.05 and **p<0.01, compared to placebo group

Between groups evaluation indicated that after intervention, the *R. khorasanicum* hydro-ethanolic extract supplementation significantly decreased the levels of FBS, total cholesterol, triglyceride, and LDL-C in the extract group compared to the placebo group (p<0.05-p<0.01) (Figure 2). However, 2hPPG, HbA1c, HDL-C, SGOT, SGPT, urea, creatinine, and urine microalbumin values did not significantly vary between the placebo and extract groups (Table 2). Moreover, the *R. khorasanicum* hydro-ethanolic extract supplementation did not significantly affect the patients' BP compared to the placebo after three months of intervention (Table 2). No adverse effects were noted by the patients of either group. 

## Discussion

Diabetes mellitus is a highly prevalent disease with long-term metabolic disturbances causing cardiovascular, neuropathic, and renal complications; however, there is still no effective medication for improving patients' life expectancy and quality of life (Garg et al., 2020 ▶). Therefore, considering alternative complementary medicine, especially medicinal plants in the management of diabetes patients is an urgent need. In recent decades, researchers have studied several herbal plants as adjunct or alternative anti-diabetic agents (Jacob and Narendhirakannan, 2019 ▶).

To the best of our knowledge, there is no clinical report about the anti-diabetic effects of *R. khorassanicum*. In this RCT, three-month co-supplementation with *R. khorassanicum *hydro-ethanolic extract showed antihyperglycemic and antihyperlipidemic effects without any side effects or alterations in liver and kidney function tests. These results supported the previous study which demonstrated that treatment of streptozocin-induced diabetic rats with *R. khorassanicum *hydro-ethanolic extract (500 mg/kg /day) decreased serum levels of glucose, TC, LDL-C, TG, SGPT, creatinine, and urea, and improved insulin levels and kidney and liver oxidative stress status. Moreover, the anti-diabetic effects of *R. khorassanicum* extract were comparable with those of metformin (500 mg/kg/day). Therefore, the anti-diabetic effects of *R. khorassanicum* might be related to its antioxidant properties and its stimulatory effects on insulin secretion (Gholamnezhad et al., 2021 ▶).

The fruit extract of *R. khorassanicum *is rich in phenolic compounds and anthocyanins; α-amylase and α-glucosidase inhibitory activities of these compounds have been reported (Da Silva Pinto et al., 2010 ▶; Grussu et al., 2011 ▶). The inhibition of these enzymes might slow starch hydrolysis which results in reducing and delaying intestinal glucose absorption (Akkarachiyasit et al., 2010 ▶; Belwal et al., 2017 ▶; Grussu et al., 2011 ▶). In addition, anthocyanin increase glucose uptake by enhancing adipose tissue and muscle sensitivity to insulin (Turrini et al., 2017 ▶). 

Although there was no significant difference in HbA1c values between the placebo and extract groups, comparison of baseline and after-treatment values showed a significant reduction in HbA1c values in the extract-treated group. HbA1c level is a reliable indicator of the glycemic index and it was also introduced as a predictor of hyperlipidemia (Hussain et al., 2017 ▶). The results of this study showed that *R. khorassanicum *co-administration could affect both glycemic index and lipid profile which are important in prognosis of cardiovascular complications. *R. khorassanicum* significantly reduced patients' blood pressure compared to the placebo. An animal study indicated that the anti-hypertensive effect of this plant was mediated through modification of nitric oxide pathway or oxidative stress status (Hamounpeima et al., 2019 ▶). Moreover, both *in vitro *and *in vivo* experiments have shown that anthocyanin inhibits the lipogenesis activity while enhances the activity of lipolytic enzymes (Belwal et al., 2017 ▶; You et al., 2011 ▶). In a clinical study, anthocyanin supplementation alleviated diabetic patients’ hyperlipidemia and decreased their serum levels of LDL cholesterol, triglycerides, apolipoprotein (apoB-48 and apoC-III) while increased HDL cholesterol, improved antioxidant capacity, and reduced insulin resistance (Li et al., 2015 ▶). Adjunct therapy with *V. arctostaphylos* (another identified taxonomic spices of Ghareghat) leaf hydro-alcoholic extract alongside conventional drugs exerted additional antihyperglycemic, antihyperlipidemic and antihypertensive effects in hypertensive type 2 diabetic patients (Mohtashami et al., 2019 ▶).

In the present study, comparison of baseline and after-treatment values showed significant improvements in FBS, 2hPPG, and diastolic and mean arterial pressure of the placebo group. Patients' participation in a study might affect psychological factors which lead to better following of the instructions and medication related to diabetes management. In this study, patients were asked to continue their routine physical activity and diet, but they were not advised to report them and it could be a limitation of this study. More clinical trials are needed before introduction of adjunct therapy with *R. khorassanicum* for ameliorating diabetes-induced metabolic disturbances in the patients. In addition, mechanisms underlying hypoglycemic and hypolipidemic effects of this plant and its constituents should be evaluated in future studies.

This study is the first clinical study about the effects of *R. khorassanicum* extract and no adverse effects were noted by the patients of the extract group. However the tolerability and safety of the *V. arctostaphylos* had been established by previous studies (Kianbakht and Hashem-Dabaghian, 2019 ▶; Mohtashami et al., 2019 ▶; Soltani et al., 2014 ▶).

In conclusion, co-supplementation of diabetic patients with *R. khorassanicum* extract ameliorated hyperglycemia and hyperlipidemia without causing adverse effects therefore, it could be recommended as an alternative therapy to improve diabetes-induced metabolic disturbances.

## Conflicts of interest

The authors have declared that there is no conflict of interest.
